# Social work and preventing and countering violent extremism (P/CVE): A scoping review protocol

**DOI:** 10.1371/journal.pone.0357887

**Published:** 2026-09-09

**Authors:** Andrew McKenzie, David Yuzva Clement

**Affiliations:** 1 Doctoral Student, Factor-Inwentash Faculty of Social Work, University of Toronto, Toronto, Ontario, Canada; 2 Adjunct Research Professor, School of Social Work, Carleton University, Ottawa, Ontario, Canada; University of Amsterdam, NETHERLANDS, KINGDOM OF THE

## Abstract

In today’s hybrid and digitally connected world, hate and violent extremism persist across social and digital environments worldwide, driven by complex radicalization pathways that emerge among individuals and groups often driven by grievance and complex vulnerability. In addition to their most violent expressions, including hate crimes, extremist and terrorist violence, these phenomena wield severe negative, detrimental, traumatic, and intergenerational impacts. As governments increasingly adopt social care and public health approaches to prevent and counter violent extremism and associated harms, a gap has emerged in understanding how social workers describe their roles, theoretical frameworks, and practice experiences in what has become known as the field of *preventing and countering violent extremism* (P/CVE). Problematically so, social work research and scholarship regarding P/CVE seems to remain limited conceptually and geographically dispersed, leading to uncertainty and practical challenges for professionals in the field. To date, no scoping review has been conducted to systematically assess, synthesize, and discuss social work research related to P/CVE. Against this backdrop, the objective of this scoping review is to identify the current research landscape, gaps, and future directions regarding social work and P/CVE. It aims to provide a comprehensive overview of elements, findings, and practice-relevant limitations and opportunities on social work and P/CVE and contributes to both the development of a nuanced understanding of an emerging social work practice field while also furthering the knowledge base and theory of P/CVE.

## Background

Across many jurisdictions, concerns about rising hate and violent extremism have intensified, driven by growing social polarization, online misinformation and hostile public discourse, but also due to increased loneliness and socioeconomic instability. Individuals often become radicalized gradually, engaging first with extremist ideas in digital ecosystems before adopting more rigid, exclusionary us-versus-them worldviews. This process can lead some people—often isolated, aggrieved, or searching for belonging—to embrace violent extremist ideologies that target perceived “enemies” or marginalized communities. ‘Violent extremism’ describes violent actions and beliefs and can be committed towards a targeted group of people for the sake of an extremist ideology [1], often in the case of so-called “homegrown terrorism” [2]. In Canada (where the authors reside), there are several notable examples of violent extremism entrenchment and offending. Almost 200 Canadians travelled to Syria to join the so-called *Islamic State* or *Daesh* [3], an example of what the Canadian government terms *religiously motivated violent extremism* [4]. The most prolific type of violent extremism in Canada currently is *ideologically motivated violent extremism*, comprising 50% of domestic terrorism cases [4]. Ideological extremist violence is driven by xenophobic ethno-nationalism, misogyny, and anti-authority/government sentiment. Instances in Canada include an ethnonationalist attack on a mosque which killed six worshippers, and a misogynist attack by a man driving a van targeting women, killing 11. The Royal Canadian Mounted Police, Canada’s federal police force, published a report along with international enforcement and intelligence allies calling for greater public awareness of the growing trend of radicalization, particularly among young people [5]. This protocol outlines the conceptual scope of prevention and intervention programs addressing violent extremism, overviews their intersections with social work, and details the methodological framework for the scoping review, which will be conducted at a subsequent stage.

### Historical development of domestic counterterrorism

In the immediate post‑9/11 era, many Western governments engaged in the “Global War on Terror,” operating under the assumption that violent extremism could be countered primarily on foreign soil through security measures. Counterterrorism during this period focused heavily on intelligence collection, military operations overseas, and domestic law enforcement-led operations. Securitization and stigmatization have been a major point of critique in the development of such counterterrorism policies. Across several national contexts, scholars and community advocates have expressed concern about how risk‑based discourses and securitized forms of governance have negatively affected already stigmatized communities, with particular attention paid to Muslim communities living in the Global North [6,7]. Such criticisms revealed the harms of a security-centric approach.

Since the 2010s North American and European governments have shifted toward whole-of-society approaches that incorporated more social care, community‑based contexts. The security paradigm still existed, but was recognized (at times) as harmful and inadequate on its own. The shift towards social care approaches prompted governments to fund research and intervention programs involving health professionals, community organizations, religious leaders, and other frontline practitioners to support individuals at risk or already engaged in violent extremism to divert them away from risk and violent acts and support social reintegration and rehabilitation. Throughout the 2010 decade, these investments gradually evolved into a distinct violence prevention field now widely recognized as preventing and countering violent extremism or P/CVE.

Often led by civil society organisations [8], P/CVE programs typically follow public health care and social work strategies, working both upstream and downstream of the issue by using primary, secondary, and tertiary interventions. Primary prevention interventions consist of community-wide outreach efforts to mitigate risk factors of extremism and enhance “community resilience.” Secondary prevention activities are directed at people who are at risk of radicalizing to violence but are not engaged and fixated on violence or planning [9]. Tertiary interventions are directed towards those who have committed or are planning extremist or terrorist violence and may unfold in criminal justice settings [10,11]. Further, such interventions typically provide services to caregivers and work alongside other professionals within multi-sectoral and interdisciplinary settings to increase protective factors and mitigate risk factors. P/CVE program development is a priority for Western democracies to enhance public safety and resilience to domestic violent extremism [12–16].

### P/CVE theory and practice

While P/CVE program development has expanded across jurisdictions, many of its core concepts remain contested. The constructs *radicalization* and *deradicalization* are often used yet increasingly critiqued for being reductionist [17], particularly for placing too much conceptual weight on the role of ideology [18]. Nevertheless, as a concept, *radicalization* is widely used and broadly defined as the pathway of a person or group adopting an ideology that promotes hatred and extremist violence and moves them toward mobilizing and committing acts of hate‑motivated, extremist, or terrorist violence. A growing body of research highlights that radicalization is rarely linear and can take multiple paths [17,19–22]. Yet one commonality across radicalization models is the understanding that it is a *process* influenced by an interplay of risk factors and stressors (e.g., trauma, discrimination, bullying, social isolation, family adversity), and exposure to and fixation towards extremist or hateful messaging. These influences interact with grievance‑based motivations (i.e., political, social, religious, ideological, or non‑ideological) and individual sociopsychological factors (e.g., mental health challenges, low self‑esteem).

The terms *deradicalization* and/or *disengagement* became the catch-all terms for the myriad processes which supported a person’s cognitive and behavioural movement away from or resilience against violent extremism [2,21]. Protective factors are numerous and can include personal factors such as strong mental health, critical thinking, and hopefulness, as well as social factors such as supportive relationships, strong community ties, school connectedness and personal/professional development trajectories. Protective factors also reduce the likelihood that individuals already affiliated with extremist networks will move from extremist beliefs to violent action [19].

An emerging area of research concerns which P/CVE practices—such as programs, interventions, and treatments—are effective in “deradicalizing” individuals at risk of engaging in violent extremist acts or pathways. There is some growing consensus on practices such as multi-agency, social re-integration approaches, which are backed by several systematic reviews [10,22]. Additional tensions persist around ethics and practice, especially the challenges that arise when human service providers are tasked to work alongside law enforcement while maintaining trust, confidentiality, and professional boundaries. At the same time, prevention efforts in digital contexts (whether broad-based or focused on individuals identified as at-risk) have evolved significantly in recent years yet still lack comprehensive evaluation findings. This gap underscores the need for ongoing research, knowledge exchange, and evidence‑informed learning across scientific communities.

Helping professions, such as social work, have the dual burden not only of contributing to the growing scholarship but directing their frontline members towards quality literature that will inform their practice. Systematized knowledge, however, on social work’s contribution and discussion of preventing and countering violent extremism (P/CVE) lack comprehensiveness. Existing scholarships are limited and scattered across different national contexts and conceptual traditions, making it difficult to form a coherent understanding of how social workers engage with this emerging area of practice. This fragmentation creates uncertainty and practical challenges for professionals who are increasingly expected to contribute to prevention and intervention efforts concerning violent extremism [23]. Notably, no scoping review has yet been conducted on this topic, leaving an important gap in understanding how social work participates in, informs, or critically interrogates P/CVE theory and practice. This scoping review will offer an in‑depth exploration of the distinct and emerging role of social work in P/CVE, highlighting its unique contributions, challenges, and opportunities.

### Social work

For the purpose of this review, social work is defined as a specific academic discipline (scholarship) and a practice-based profession (practice) committed to equity, social justice and social change. We define social work scholarship as academic research and studies carried about by members (e.g., academics, researchers, lecturers, graduate students) affiliated with a university faculty of social work or who self-identify as social work scholars. Social work practitioners hold university training in social work and use their skills to serve children, youth, adults including older adults, families, groups and communities as well as larger social or organizational systems. Social workers utilize theory to inform practices that enhance individual and collective well-being, social justice, and dignity while simultaneously considering and critically examining people’s individual problems and broader socio-economic, historical, political and environmental issues and structures [24,25].

Our definition of social work encompasses a broad practice internationally. Relevant to our study is the difference between Germany and English-speaking nations. For instance, in Germany social work encompasses youth workers as well as child and youth care workers, community development workers/community liaison officers, community workers, welfare officers, and social/cultural animators. In North America, however, there are distinct disciplines or programs of study for child and youth work. Also, in Germany social workers cannot provide clinical psychotherapy, though in North America this training is provided at the master’s level.

Social work academics, frontline practitioners and institutional leaders are becoming increasingly involved in P/CVE as part of the whole-of-society approach to preventing extremist radicalization [5,26]. Social work involvement in P/CVE may be in the form of programming that specializes in violent extremism prevention, often funded directly by government ministries oriented towards public safety. Such programming may include standalone, localized programs [20], or wide ranging national initiatives tied to legal mandates on social workers such the Prevent mandate in the United Kingdom [27]. Social workers performing in standard practice domains (e.g., child protection, school-based, family services, corrections, housing support) can also encounter clients that are involved in some form of hate-based or extremist ideology or support caregivers whose loved ones have become involved in violent extremism pathways [28,29].

### Social work tensions

It is important to recognize that social work has drawn attention to concerns that P/CVE is, at its roots, a form of securitization that may co-opt the profession into a repressive function [30–32]. Scholars have cautioned that a “move into… counterterrorism work, is something that social work practitioners should not accept uncritically, or even possibly at all” [33]. For example, research on youth work in the Netherlands found that youth workers engaged in forms of ethical trespass, including the stigmatization of youth, breach of trust, and neighbourhood surveillance [34]. Similarly, researchers in Norway documented instances in which social workers operated within a blurred boundary between support and policing, particularly when practices encroached upon clients’ rights to privacy and confidentiality [23]. These findings demonstrate how professionals may become implicated in securitized practices when critical reflection on their role and mandate is lacking. They also expose the limitations of approaches that construct radicalization primarily as a youth problem, neglecting its broader and more complex social etiology. There is an urgent need to integrate these critiques into social work practice and knowledge development if the profession is to maintain its ethical commitment to human rights, social justice, and emancipation.

### Study objectives

To date, a comprehensive framework to guide social workers engaged in P/CVE remains absent. There are several systematic evidence reviews on P/CVE—including on interventions, case management, and multi-sectoral collaboration— that certainly overlap with social work competencies [10,22], and are helpful in demonstrating the relevance of the profession. However, there are no reviews that include conceptual, practical, and ethical guidance specifically for social work practice and education, leaving a significant knowledge gap.

Although social work scholarship has generated some research on P/CVE, it is fragmented and lacks systematic organization. This is partly an effect of the early stage of P/CVE development, as the field is only about a decade old. As the field matures, there lies the opportunity for social work scholarship to strengthen its contributions by bringing together and building on the early body of work and pointing to both gaps and future orientation.

The primary objective of this scoping review is to synthesize the general themes across the social work literature on P/CVE, with attention paid to how scholars conceptualize the particular role of social work in P/CVE, including the relevant theoretical perspectives. This general review will provide a theoretical foundation from which future scholars can refer.

The second objective is to synthesize specific recommendations for social work educators and practitioners in P/CVE. Complex practice domains, such as child welfare or suicide prevention, will have entire textbooks devoted to guiding students towards evidence-based or -informed practices. The current knowledge gap in radicalization is particularly problematic as it can lead to—as discussed above—inconsistent responses and ethical dilemmas for practitioners. This scoping review will synthesize the available knowledge to date, including theoretical and empirical perspectives, and be useful to practitioners who need a social work-based resource to inform their practice decisions.

## Materials and methods

This scoping review will follow the five-stage framework proposed by Arksey and O’Malley [35], which is further improved by Levac and colleagues [36] and the Joanna Briggs Institute [37]. This methodology is the most appropriate given the nascent nature of social work scholarship concerning P/CVE, and the need to start by systematically mapping the literature to date. Reporting will also conform to the Preferred Reporting Items for Systematic Reviews and Meta-Analysis: Extension for Scoping Reviews (PRISMA-ScR) as recommended for methodological rigor (see S1 Checklist) [38].

The scoping review team includes two social workers. One is fluent in German and English (DYC) while the other is fluent only in English (AM). Each has a somewhat different social work history. The German-speaking team member practiced primarily as a community social worker in Germany prior to moving to Canada. He works in public policy related to P/CVE and is an adjunct research professor at a Canadian university. The English-only team member practiced as a frontline social worker for over a decade, with five years in the P/CVE field before pursuing his doctoral studies at a Canadian university.

### Stage 1: Identify the research questions

The overarching research question guiding this scoping review is: What is the landscape of social work within or concerning the field or practice of P/CVE? Specifically, the three sub-research questions are:

a. What themes and theories are present in current social work literature on P/CVE?
b. What issues are identified for social workers in P/CVE, and
c. What guidance is provided to support social work practice in or regarding P/CVE?

### Stage 2: Identify relevant literature

This review will include publications in English and German. The four English databases are as follows: APA PsychInfo (Ovid platform), Social Work Abstracts (Ovid platform), Scopus, and Criminal Justice Abstracts (EBSCO platform). The four German databases will be Social Science Open Access Repository (SSOAR) (GESIS Search platform), KrimDok, Bielefeld Academic Search Engine (BASE), and K10plus.

The scoping review is limited to English as it is the most prolific language in the topic, and German, given that Germany has a long and robust history of publishing in this area and leaving out German-only literature would not reflect the historical development of the P/CVE field.

This review combines two concepts: 1) P/CVE, and 2) social work. The first concept, P/CVE, is an emerging acronym that is not yet robustly used across all fields, and so other associated concepts will be used such as counterterrorism, deradicalization, and extremism. The second concept, social work, is a robust terminology across domains. Related terminology, such as case management, youth work, and social services, will be used as well. The review will search for terms in title, abstract, headings and author-assigned keywords or key concepts. The PsychInfo (Ovid) search string is included in S2 Table and will be the basis for translation into other database platforms.

Grey literature will be limited to the following publications: 1) national or federal government sponsored evaluations of P/CVE programs which include national social work and social work college or association publications related to P/CVE. Government, professional college or association publications at the regional (e.g., state, province) and municipal levels will be excluded. The following P/CVE nongovernmental organizations are well recognized in the field and will be searched for publications related to social work: the Centre for Research and Evidence on Security Threats (CREST), the Prevention Practitioners Network (PPN), the Canadian Practitioners Network for the Prevention of Extremist Violence (CPN-PREV), the Addressing Violent Extremism and Radicalisation to Terrorism (AVERT) Research Network, RAND (the RAND Corporation) and the European Union Knowledge Hub on Prevention of Radicalisation and its precursors.

The inclusion criteria are: 1) peer-reviewed academic literature, inclusive of commentaries, theoretical papers and empirical studies; 2) books and/or dissertations; 3) geographically situated in North America, Europe, United Kingdom (UK), New Zealand or Australia; 4) includes mention of P/CVE and social work; 6) publications written in English or German; and 7) publications dated January 1^st^, 1990 or later. Publications are not limited to empirical studies because the reviewers include synthesizing the landscape of social work thought and opinion regarding P/CVE. Commentary (e.g., position papers), practitioner books and early career dissertations form an important portion of the published landscape to date.

The above geographic limitations align to an international P/CVE community of practice. These countries participate in collaborative research, cross-border funding, information sharing, and regular conferences. Aligning the scoping review with the community of practice helps to ensure the constructs (e.g., ‘violent extremism’ and ‘social work’) are consistent within publications.

Regarding German full-text publications, the one bilingual (German–English) researcher on the team (David Yuzva Clement or DYC) will be the primary reviewer for the German-language articles. Given that the other researcher (Andrew McKenzie or AM) does not speak German, both researchers will regularly meet to discuss (in English) each article written in German. Screening decisions for German-language material will be discussed in English during consensus meetings, consistent with evolving standards for language-expert screening within a dual independent review [39–41].

Regarding dates, while the September 11, 2001 terrorist attacks against the United States marked a major shift in counterterrorism and prevention policy and practice in North America, Germany had a robust debate that dates back to the 1990s. Germany’s post-WWII criminalization of Nazism and neo-Nazism made it an early leader in P/CVE practice, and so a cut off date of January 1, 1990, will set the scope of publications and a more equal basis for cross-national comparison.

### Stage 3: Literature selection

The researchers will input all literature retrieved from the above databases using Covidence software to deduplicate and facilitate the screening process. Firstly, two researchers (AM/DYC) will independently screen all titles and abstracts against inclusion criteria and record their decisions on Covidence. The researchers will re-calibrate their criteria if their agreement rate is under 80% after the first 50 text screenings.

Following title and abstract screening, researchers will independently conduct a full-text review of each accepted document [42]. Any disagreements will be resolved through a discussion and consensus model, with third-party support if needed. All decisions and reasons for exclusion at the full-text stage will be documented to support transparency and align with scoping review reporting standards. The literature selection process will be narrated and reported using a PRISMA flow chart.

### Stage 4: Charting the data

The data extraction process will be calibrated on 10 publications, divided into 5 empirical studies with human participants and 5 publications that are either theoretical or study non-human subjects (e.g., discourse analysis) [36]. Of the 10 publications, at least one must be in German. The initial data extraction form, produced in Microsoft Excel, will include the following sections: Author, year, publication type (e.g., peer-reviewed journal article, government sponsored publication), publication objective or study research question, study characteristics (e.g., type, year, location, funding source, P/CVE context), study methodology (e.g., theory, sample, design, *n*), findings and/or author points. During this calibration stage the researchers will decide whether the initial extraction form can be adapted to fit all publication types, or whether multiple forms or classifications should be used according to study type [36].

After calibration and extraction form creation, researchers will progress through selected publications in the same sequence, meeting approximately once per month or after every twenty studies. These meetings will include discussing discrepancies, adjusting forms if needed, and the presentation of German articles to the native English reviewer. Discrepancies that cannot be resolved will be referred to a social work professor (or otherwise) familiar with scoping reviews. The final extraction form(s) will be included in the appendix.

### Stage 5: Collating, summarizing, and reporting the results

The heterogeneity of included publications means that researchers cannot know the exact format for collating and reporting the results [37]. Give the large representation of qualitative studies, the best course of action appears to be a narrative report organized by theme. The authors will engage in thematic analysis to move from mere description of the data towards broader conceptual conclusions about social work practice in P/CVE. This form of six-stage thematic analysis in the tradition of Braun and Clarke [36,43] will be facilitated by NVivo software [44]. Given the research question seeks to survey the landscape of social work in and concerning P/CVE, a visualization component will likely be used after the narrative reporting to provide an overview of social work theory within the context of country and P/CVE program type. Most likely, this visualization will take the form of a conceptual model or continuum. Regarding empirical literature, a table of all included studies will be presented in the appendix. Table columns will include citation information, study location, sample, research question or purpose, study design, summary findings and limitations.

### Stage 6: Consultation

Researchers will present their preliminary findings to stakeholders in the form of two online webinars, one in English and one in German. The webinar is also a form of knowledge transfer, facilitating the utility of this scoping review to the field [36]. Invitations will be sent to P/CVE practitioner networks in North America, the UK and Europe, as well as social work educators who have published on P/CVE. Feedback will be requested in the second half of the webinar, and an online form will be shared to allow for written feedback. The researchers may use the findings of the consultation within the scoping review.

## Discussion

The purpose of the scoping review is to summarize the state of social work theory and practice within and concerning the context of preventing and countering violent extremism (P/CVE). Given this wide scope, neither the quality of studies or study author bias will be assessed [35,36,45]. The inclusion of grey literature and books introduces a further limitation in quality. The researchers will also include a position statement as to their professional roles as social workers within the P/CVE field.

The researchers chose to adopt such a wide scope (even with limitations) in order to provide what may be the first scoping review of social work and P/CVE. The overarching objective is to provide a timely and foundational contribution that advances both social work and P/CVE, while outlining substantial implications for theory, research, and practice development within the nexus of social work and P/CVE. Future researchers, but also social workers and other frontline practitioners, will have a single, summative resource grounded in rigorous methodology from which to launch further inquiry.

## Dissemination

We will disseminate the findings through publication in a peer-reviewed journal. In addition, we will develop a concise briefing document that summarizes key results and implications for social work education and practice, tailored for social work educators and practitioners. To support uptake and discussion, we will host a webinar to share the briefing, highlight practical takeaways, and engage stakeholders in interpreting how the findings can inform curriculum development, training, and practice guidance.

## Limitations

This review focuses on a defined set of countries and organizations to enable a manageable scope and clearer cross-national comparison; however, this geographic focus excludes social work scholarship from the Global South where domestic extremist violence is also being addressed. In addition, the grey literature sampling strategy privileges well-established and widely recognized P/CVE nonprofits and networks, which may result in missed publications from newer or less visible organizations that have not yet achieved comparable recognition. Our emphasis on federal governments and national social work associations may also underrepresent subnational or regional policy and practice literature, including publications produced by provinces, states, municipalities, and local professional bodies. Together, these design choices may limit the completeness of the mapped evidence base and may bias findings toward jurisdictions and institutions with greater capacity for formal publication and dissemination.

## Supporting information

S1 Checklist PRISMA-ScR (Preferred Reporting Items for Systematic Review and Meta Analysis Protocols) checklist adapted for the scoping review protocol. (DOCX)

S2 Table Search string for PsycInfo as a pilot database. (DOCX)
