# Supplementary material for: Social work and preventing and countering violent extremism (P/CVE): A scoping review protocol
Source: PLoS One. 2026 Sep 9;21(9):e0357887. doi: 10.1371/journal.pone.0357887 (PMC13557355; doi:10.1371/journal.pone.0357887)
Supplement: Supplementary file 2 [file pone.0357887.s002.docx]

**S2Table: Search string for PsycInfo as a pilot database.**

| APA PsycInfo <January 1, 1990 to October 2026> | Search String |
| --- | --- |
| 1 | social services/ or community services/ or outreach programs/ |
| 2 | social casework/ or social group work/ |
| 3 | social workers/ or psychiatric social workers/ |
| 4 | social work education/ |
| 5 | (social work* or youth work* or social casework* or "community work*" or MSW or BSW).tw. |
| 6 | 1 or 2 or 3 or 4 or 5 |
| 7 | exp Extremism/ or exp Counterterrorism/ or exp Terrorism/ |
| 8 | ("countering violent extremism" or "preventing violent extremism" or "CVE" or "PVE" or "P/CVE").mp. [mp=title, abstract, heading word, table of contents, key concepts, original title, tests & measures, mesh word] |
| 9 | (counterterroris* or counter-terrorism or terroris* or counter terrorism or (extremis* or radicaliz* or radicalis* or deradicali*) or (jihadism or daesh or ISIS or AQ) or (far-right or alt-right or alt right) or (far-left or left-wing) or (incel or manosphere) or (neo-nazi* or ethnonation*) or nihilis* violence) or (anti-authority extremis*) or (anti-institutional extremis*)).ab,hw,id,mf,ot,tc,ti,tm. |
| 10 | 7 or 8 or 9 |
| 11 | 6 and 10 |

PsychInfo search string ‘translated’ into other platforms

| Worldwide Political Science Abstracts: | Search String |
| --- | --- |
|  | (MAINSUBJECT.EXACT("Social work") OR MAINSUBJECT.EXACT("School social work") OR MAINSUBJECT.EXACT("Social services") OR MAINSUBJECT.EXACT("Community services") OR MAINSUBJECT.EXACT("Outreach programs") OR MAINSUBJECT.EXACT("Social workers") ) OR TIAB("social work*" OR "youth work*" OR "social casework*" OR "community work*" OR MSW OR BSW) |
| AND | (MAINSUBJECT.EXACT.EXPLODE("Terrorism") OR MAINSUBJECT.EXACT("Extremism") OR MAINSUBJECT.EXACT("Counterterrorism")) OR TIABSU("countering violent extremism" OR "preventing violent extremism" OR CVE OR PVE OR P/CVE) OR TIABSU(counterterroris* OR counter-terrorism OR terroris* OR "counter terrorism" OR (extremis* OR radicaliz* OR radicalis* OR deradicali*) OR (jihadism OR daesh OR ISIS OR AQ) OR (far-right OR alt-right OR "alt right") OR (far-left OR left-wing) OR (incel OR manosphere) OR (neo-nazi* OR ethnonation*) OR (nihilis* violence) OR (anti-authority extremis*) OR (anti-institutional extremis*)) |

| Scopus | Search String |
| --- | --- |
| N/A | ( TITLE-ABS-KEY ( ( "social work*" OR "youth work*" OR "social casework*" OR "community work*" OR MSW OR BSW ) ) AND TITLE-ABS-KEY ( ( "countering violent extremism" OR "preventing violent extremism" OR CVE OR PVE ) OR ( counterterroris* OR counter-terrorism OR terroris* OR "counter terrorism" OR ( extremis* OR radicaliz* OR radicalis* OR deradicali* ) OR ( jihadism OR daesh OR ISIS OR AQ ) OR ( far-right OR alt-right OR "alt right" ) OR ( far-left OR left-wing ) OR ( incel OR manosphere ) OR ( neo-nazi* OR ethnonation* ) OR (nihilis* violence) OR (anti-authority extremis*) OR (anti-institutional extremis*)) ) ) |
